# Targeting metabolic pathways alleviates bortezomib-induced neuropathic pain without compromising anticancer efficacy in a sex-specific manner

**DOI:** 10.3389/fpain.2024.1424348

**Published:** 2024-06-24

**Authors:** Panjamurthy Kuppusamy, Md Mamunul Haque, Richard J. Traub, Ohannes K. Melemedjian

**Affiliations:** ^1^ Department of Neural and Pain Sciences, University of Maryland School of Dentistry, Baltimore, MD, United States; ^2^ UM Center to Advance Chronic Pain Research, Baltimore, MD, United States; ^3^ UM Marlene and Stewart Greenebaum Comprehensive Cancer Center, Baltimore, MD, United States

**Keywords:** chemotherapy-induced peripheral neuropathy (CIPN), bortezomib, metabolic interventions, metformin, dichloroacetate, oxamate, Lewis lung carcinoma (LLC), sex differences

## Abstract

**Introduction:**

Chemotherapy-induced peripheral neuropathy (CIPN) is a debilitating side effect of cancer treatment that significantly impacts patients' quality of life. This study investigated the effects of targeting metabolic pathways on bortezomib-induced neuropathic pain and tumor growth using a Lewis lung carcinoma (LLC) mouse model, while exploring potential sex differences.

**Methods:**

Male and female C57BL/6J mice were implanted with LLC cells and treated with bortezomib alone or in combination with metformin, dichloroacetate (DCA), or oxamate. Tactile allodynia was assessed using von Frey filaments. Tumor volume and weight were measured to evaluate tumor growth.

**Results:**

Metformin, DCA, and oxamate effectively attenuated bortezomib-induced neuropathic pain without compromising the anticancer efficacy of bortezomib in both male and female mice. The LLC model exhibited a paraneoplastic neuropathy-like phenotype. Significant sex differences were observed, with male mice exhibiting larger tumors compared to females. Oxamate was more effective in alleviating allodynia in males, while metformin and DCA showed greater efficacy in reducing tumor growth in females.

**Discussion:**

Targeting metabolic pathways can alleviate CIPN without interfering with bortezomib's anticancer effects. The LLC model may serve as a tool for studying paraneoplastic neuropathy. Sex differences in tumor growth and response to metabolic interventions highlight the importance of considering sex as a biological variable in preclinical and clinical studies investigating cancer biology, CIPN, and potential therapeutic interventions.

## Introduction

Chemotherapy-induced peripheral neuropathy (CIPN) is a debilitating side effect of various chemotherapeutic agents, including the proteasome inhibitor bortezomib. CIPN affects up to 80% of patients who receive anticancer therapy and is characterized by severe pain and sensory disturbances that can persist even after the cessation of chemotherapy, significantly impacting patients' quality of life. This adverse effect can be severe enough for patients to either reduce the dosage of anticancer treatment or stop the treatment altogether, compromising the effectiveness of cancer therapy (1–3). Unfortunately, current treatment options for CIPN are vastly inadequate, and there is a pressing need for the development of novel therapeutic strategies that can alleviate CIPN without interfering with the anticancer effects of chemotherapeutic agents.

Recent studies have shown that bortezomib induces metabolic reprogramming in sensory neurons, leading to a shift towards aerobic glycolysis (4), a metabolic phenotype commonly observed in cancer cells (5). This alteration in cellular metabolism has been linked to the development and maintenance of bortezomib-induced neuropathic pain. Specifically, bortezomib stabilizes the expression of hypoxia-inducible factor 1α (HIF1A) (6), a transcription factor that regulates the expression of genes involved in glycolysis and energy production under hypoxic conditions. The stabilization of HIF1A leads to the upregulation of pyruvate dehydrogenase kinase 1 (PDHK1) and lactate dehydrogenase A (LDHA), two key enzymes that maintain aerobic glycolysis (4).

PDHK1 phosphorylates and inhibits pyruvate dehydrogenase (PDH), attenuating mitochondrial pyruvate oxidation and promoting the conversion of pyruvate to lactate by LDH. This reaction regenerates the cofactor nicotinamide adenine dinucleotide (NAD+) in the cytosol, which is critical for sustaining glycolysis. The resulting lactate is then extruded from the cell along with a proton, leading to increased extracellular acidification, which has been implicated in the development and maintenance of CIPN (4).

Interestingly, aerobic glycolysis is a common metabolic phenotype observed in cancer cells (5), and targeting this pathway has been shown to inhibit tumor growth (7–24). Metformin, a widely used antidiabetic drug, has been shown to inhibit the expression of HIF1A in sensory neurons, thereby attenuating the initiation of CIPN (6). Dichloroacetate (DCA), a small molecule inhibitor, targets PDHK, thereby activating PDH and promoting the entry of pyruvate into the mitochondrial Krebs cycle. This shifts the metabolism away from aerobic glycolysis and towards oxidative phosphorylation thus alleviating CIPN. Oxamate, an analog of pyruvate, inhibits lactate dehydrogenase, thereby reducing the production of lactate and attenuating the pain-inducing effects of extracellular acidification (25) in mice with bortezomib-induced neuropathic pain (4).

Given the metabolic similarities between cancer cells and neurons affected by CIPN, targeting aerobic glycolysis represents an attractive strategy for alleviating CIPN without interfering with the anticancer effects of bortezomib. In this study, we sought to investigate the effects of targeting metabolic pathways on bortezomib-induced neuropathic pain and tumor growth using a Lewis lung carcinoma (LLC) implanted mouse model. Furthermore, we aimed to explore potential sex differences in tumor burden and the efficacy of metabolic interventions in alleviating pain and reducing tumor growth.

## Materials and methods

### Study approval

All the experiments were approved by the Institutional Animal Care and Use Committee of the University of Maryland (AUP-00000034). All procedures were conducted in accordance with the Guide for Care and Use of Laboratory Animals published by the National Institutes of Health and the ethical guidelines of the International Association for the Study of Pain.

### Cell culture and reagents

Mouse Lewis lung carcinoma (LLC), cells were obtained from American Type Culture Collection (ATCC; CRL-1642, Manassas, VA, USA) and cultured in ATCC-formulated Dulbecco's Modified Eagle's Medium (DMEM; Catalog no. 30-2002; ATCC, USA), supplemented with 10% heat-inactivated fetal bovine serum (FBS; Catalog no.30-2020, ATCC, USA) and 1 U/ml penicillin-streptomycin (Catalog no.30-2300, ATCC, USA), L-Glutamine Solution(Catalog no. 30-2214, ATCC, USA) under an atmosphere of 5% CO2/95% air at 37°C and 5% CO2 in an incubator (Thermo Electron corporation, Forma Series II, Water Jacketed CO2 incubator, USA) for 48–72 h and medium was replaced by fresh medium regularly every two to three days. LLC1 cells at 70%–80% confluence, the cells were washed with 1X phosphate buffered saline pH 7.4 (PBS; Gibco, 10010056) and then treated with 0.25% Trypsin-0.53 mM EDTA solution (Trypsin-EDTA-1X; Catalog no. 30-2101) at 37°C for 3 min.

### Experimental animals and tumor implantation

Pathogen-free, 18–23 gram adult male and female C57BL/6J mice were obtained from Jackson Laboratories were housed in temperature (23 ± 3°C) and light (12-h light/12-h dark cycle; lights on 07:00–19:00) controlled rooms with standard rodent chow and water available *ad libitum*. Animals were randomly assigned to treatment or control groups for the behavioral experiments. Animals were housed five per cage. All behavioral experiments were performed by experimenters who were blinded to the experimental groups and treatments. LLC cells were cultured in an incubator at 37°C with 5% carbon dioxide and 95% air and passed twice prior to tumor implantation. Tumor growth was initiated by 1 × 10^6^ LLC cells were resuspended in 100 µl 1:1 ratio mixture of Matrigel (BD Matrigel™ Basement Membrane Matrix, BD Matrigel Matrix High Concentration; Catalog no.354248, BD Biosciences, USA) and Dulbecco's phosphate buffered saline (DPBS) were subcutaneously injected into the right inner thigh or the right side of the lower back of the C57BL/6J mice. For the implantations, mice were briefly placed under anesthesia with 1%–3% isoflurane. The mice fully recover from the anesthesia within 3 min. For baseline tactile withdrawal thresholds of the left hind paw were measured prior to the tumor implantation. Starting on day 7, the tactile withdrawal thresholds were tested. Tumor growth was monitored by measuring the perpendicular diameters (length/width) of tumor size by using a digital vernier caliper (0–200 mm) for every 2 or 3 days, and the tumor volume was calculated using the formula [Volume = (length × width^2^)/2]. On day 16, the mice were euthanized with inhaled anesthetic overdose followed by cervical dislocation. The tumors were then harvested, and weights measured.

### Drug treatments

In all the experiments and on the day of the tumor implantation C57Bl/6J mice were treated with intraperitoneal (IP) injections of 0.2 mg/kg/day(4, 6) of bortezomib (Millipore Sigma, Cat # 5.04314.0001) for total dose of 1 mg/kg, Oxamate [500 mg/kg/day(4), Millipore Sigma, Cat # O2751], dichloroacetate [100 mg/kg/day(4), DCA, Millipore Sigma, Cat # 347795] and metformin [150 mg/kg/day(6), Axxora, Cat # LKT-M2076] for five consecutive days either in combination or alone. The vehicle for bortezomib, metformin, oxamate and DCA was saline. The vehicle-treated group received intraperitoneal saline for five consecutive days.

### Mechanical testing

Male or female C57Bl/6J mice were placed in acrylic boxes with wire mesh floors, and baseline mechanical withdrawal thresholds of the left hind paw were measured after habituation for 1 h using the up-down method (26). Briefly, mice were placed in acrylic boxes with wire mesh floors and allowed to habituate for 1 h before testing. A series of calibrated von Frey filaments were applied perpendicularly to the plantar surface of the left hind paw, with sufficient force to bend the filament for approximately 2–3 s. The 50% paw withdrawal threshold was determined using the up-down method, with a minimum of 5 responses recorded for each animal. Testing was performed at baseline (before tumor implantation) and on days 7, 9, 11, and 14 post-implantation. All testing was conducted by an experimenter blinded to the treatment groups.

### Statistical analysis and data presentation

Data are based on the means and the standard error of the means (±SEM). Graph plotting and statistical analysis used GraphPad Prism Version 10 (Graph Pad Software, Inc. San Diego, CA, USA). When analyzing evoked pain behavior or tumor growth data, using two-way repeated-measures (RM) analysis of variance (ANOVA) followed by *post-hoc* pairwise comparisons with Dunnett correction was used. Tumor weights and sex differences were analyzed by one-way ANOVA followed by Tukey correction for multiple comparisons. Unpaired Student *t*-test was used to compare tumor volume and weight differences between sexes. *a priori* level of significance at a 95% confidence level was considered at *P* < 0.05.

Schematic was Created with BioRender.com.

## Results

### Metformin and aerobic glycolysis inhibitors attenuate bortezomib-induced neuropathic pain without compromising anticancer efficacy in male tumor-bearing mice

**Figure 1:**
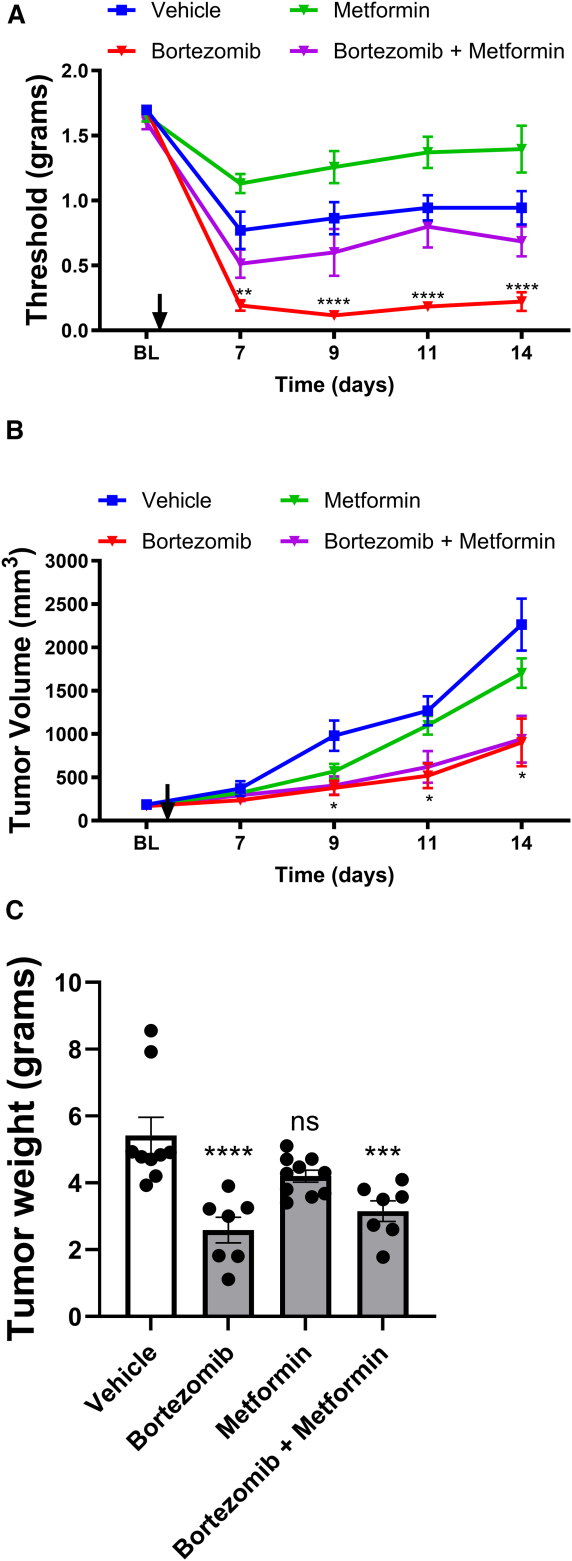
Effect of bortezomib and metformin on tactile allodynia, tumor volume, and tumor weight in male mice. (**A**) Tactile allodynia assessed using von Frey filaments at baseline (BL) and on days 7, 9, 11, and 14 post-implantation. (**B**) Tumor volume measured on the indicated days. (**C**) Tumor weight determined on day 16. (*n* = 10 mice per group). (*p < 0.05, ***p < 0.001, ****p < 0.0001, ns = not significant, vehicle vs. other groups).

The Lewis lung carcinoma mouse model, which mimics human non-small cell lung cancer (NSCLC), was employed to investigate the effects of bortezomib and metformin on tactile allodynia, tumor volume, and tumor weight (Figure 1). This syngeneic tumor model allows for the study of CIPN in immunocompetent mice (27), recapitulating the clinical scenario of NSCLC patients who often undergo surgery as a first-line treatment and receive chemotherapy to eliminate metastases (28). Furthermore, bortezomib which is commonly used to treat multiple myelomas has also been demonstrated to be effective in treating NSCLCs (29–31).

In male mice, tactile allodynia assessment (Figure 1A) revealed that the presence of the tumor itself reduced tactile thresholds, suggesting the development of paraneoplastic neuropathy-like phenotype. Metformin alone did not attenuate the tumor-induced reduction of thresholds. Bortezomib treatment produced profound allodynia; however, consistent with previous results, this chemotherapy-induced neuropathic pain was effectively reversed by metformin co-treatment.

Tumor volume measurements (Figure 1B) and tumor weight data (Figure 1C) in male mice demonstrated that while metformin alone did not significantly alter tumor growth, bortezomib treatment effectively reduced both tumor volume and weight. Importantly, metformin co-treatment did not interfere with the anticancer effects of bortezomib, as evidenced by the similar tumor growth curves and tumor weights between the bortezomib and bortezomib + metformin groups. These findings suggest that metformin may be a suitable adjuvant therapy for managing CIPN without compromising the anticancer efficacy of bortezomib.

To address the potential impact of tumor implantation site on mechanical sensitivity results, we performed additional experiments in which LLC cells were injected subcutaneously into the back of male mice (Supplementary Figure S1). Consistent with our findings from tumors implanted in the inner thigh, the presence of the tumor on the back induced a reduction in tactile thresholds, indicating the development of a paraneoplastic neuropathy-like phenotype (Supplementary Figure S1A). Bortezomib treatment significantly increased tactile allodynia compared to vehicle-treated mice, while metformin alone did not affect mechanical allodynia. Importantly, metformin co-treatment effectively blocked the bortezomib-induced tactile allodynia, demonstrating its ability to prevent the development of CIPN. Tumor volume measurements (Supplementary Figure S1B) showed that both bortezomib and bortezomib + metformin treatments significantly reduced tumor growth compared to vehicle-treated mice. These results mirror our findings from tumors implanted in the inner thigh, suggesting that the implantation site does not significantly influence the outcomes of our study. The similarity in behavioral and tumor growth patterns between the two implantation sites strengthens the validity of our findings and highlights the robustness of the LLC mouse model in studying CIPN and cancer-induced neuropathy.

**Figure 2:**
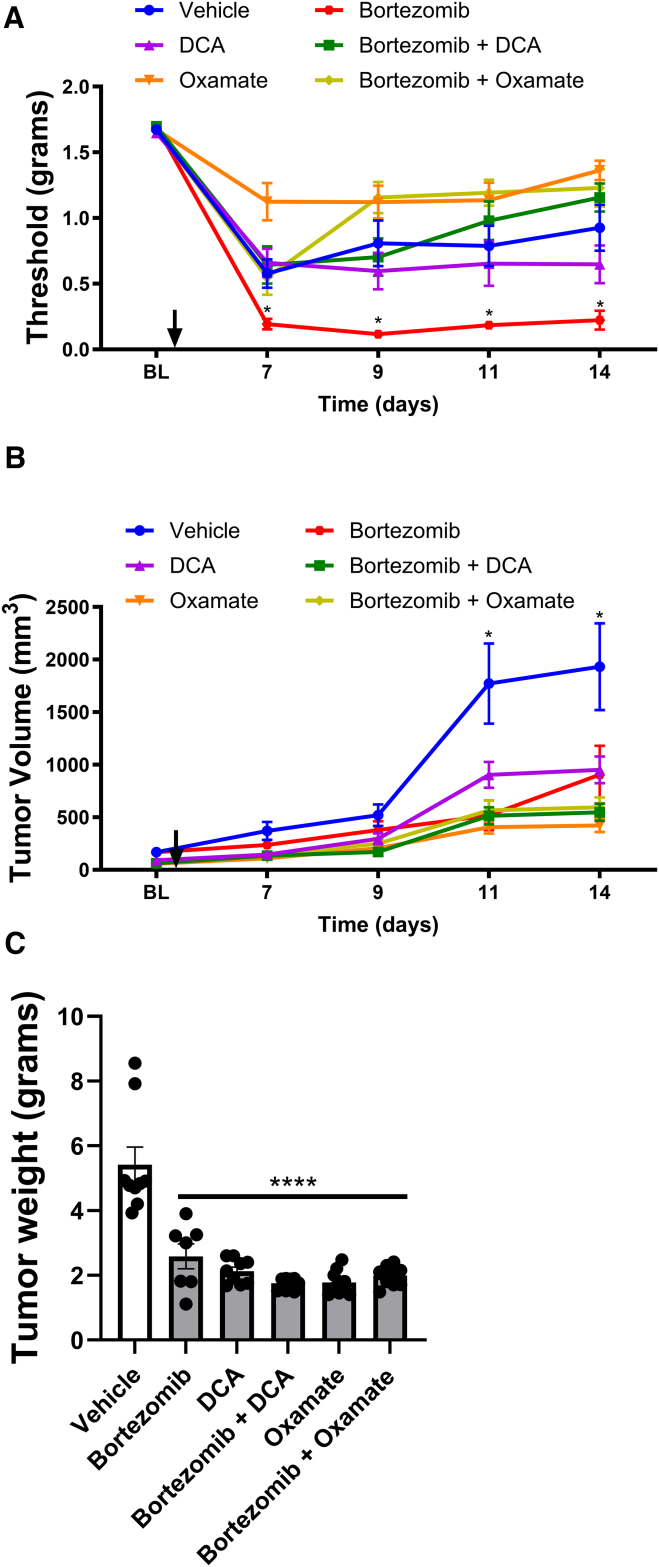
Effect of bortezomib, DCA, and oxamate on tactile allodynia, tumor volume, and tumor weight in male mouse. (**A**) Tactile allodynia assessed using von Frey filaments at baseline (BL) and on days 7, 9, 11, and 14 post-implantation. (**B**) Tumor volume measured on the indicated days. (**C**) Tumor weight determined at the end of the study (*n* = 10 mice per group). (*p < 0.05, ****p < 0.0001, vehicle vs. other groups).

To investigate the effects of targeting aerobic glycolysis on bortezomib-induced neuropathic pain and tumor growth, male mice with LLC implantation were treated with bortezomib, DCA, oxamate, or a combination of bortezomib with either DCA or oxamate (Figure 2).

Tactile allodynia assessment (Figure 2A) revealed that bortezomib treatment significantly increased tactile allodynia compared to vehicle-treated male mice, consistent with the findings in Figure 1A. While DCA and oxamate alone did not affect mechanical allodynia relative to the vehicle-treated group, co-treatment with either agent significantly attenuated bortezomib-induced tactile allodynia.

Tumor volume measurements (Figure 2B) and tumor weight data (Figure 2C) showed that treatment with bortezomib, DCA, oxamate, or a combination of bortezomib with either DCA or oxamate significantly reduced both tumor volume and weight compared to vehicle-treated male mice. Notably, the anticancer effects of bortezomib were maintained when co-administered with either DCA or oxamate, as evidenced by the similar tumor growth curves and tumor weights between the bortezomib, bortezomib + DCA, and bortezomib + oxamate groups. These findings suggest that targeting aerobic glycolysis with DCA or oxamate can complement the anticancer efficacy of bortezomib in male tumor-bearing mice.

In summary, the results demonstrate that metformin (Figure 1) and targeting aerobic glycolysis with DCA or oxamate (Figure 2) can effectively alleviate chemotherapy-induced neuropathic pain without interfering with the anticancer effects of bortezomib in male mice bearing LLC tumors. These findings highlight the potential of metformin and inhibiting aerobic glycolysis as promising therapeutic strategies for managing CIPN.

### Metformin and aerobic glycolysis inhibitors attenuate bortezomib-induced neuropathic pain without compromising anticancer efficacy in female tumor-bearing mice:

**Figure 3:**
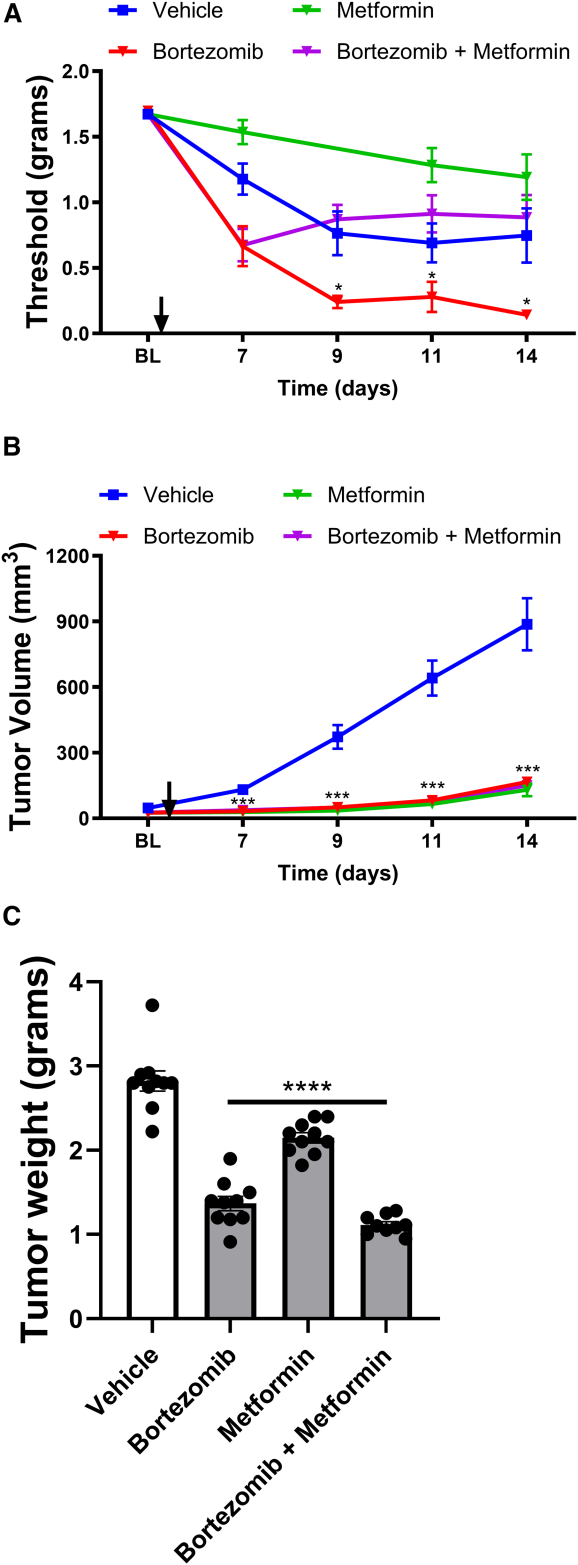
Effect of bortezomib and metformin on tactile allodynia, tumor volume, and tumor weight in female mice. (**A**) Tactile allodynia assessed using von Frey filaments at baseline (BL) and on days 7, 9, 11, and 14 post-implantation. (**B**) Tumor volume measured on the indicated days. (**C**) Tumor weight determined on day 16. (*n* = 10 mice per group). (*p < 0.05, ***p < 0.001, ***p < 0.001, ****p < 0.0001, vehicle vs. other groups).

**Figure 4:**
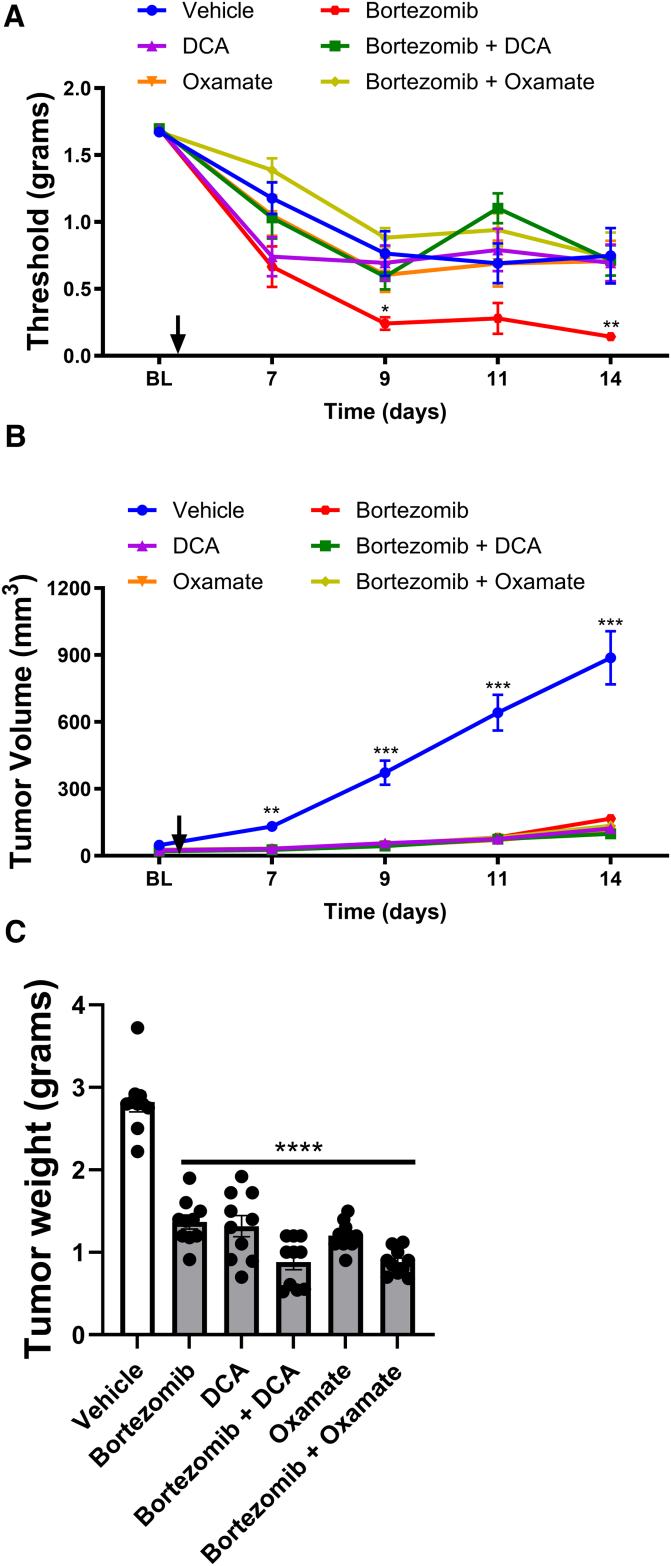
Effect of bortezomib, DCA, and oxamate on tactile allodynia, tumor volume, and tumor weight in female mice. (**A**) Tactile allodynia assessed using von Frey filaments at baseline (BL) and on days 7, 9, 11, and 14 post-implantation. (**B**) Tumor volume measured on the indicated days. (**C**) Tumor weight determined at the end of the study. (*n* = 10 mice per group). (*p < 0.05, **p < 0.01, ***p < 0.001, ****p < 0.0001, vehicle vs. other groups).

To investigate whether the effects of metformin and aerobic glycolysis inhibitors on bortezomib-induced neuropathic pain and tumor growth observed in male mice were consistent in female mice, we employed the same LLC mouse model and experimental design (Figures 3, 4).

In female mice, the presence of the tumor also led to the development of a paraneoplastic neuropathy-like phenotype, as evidenced by the reduction in tactile thresholds (Figure 3A). Metformin alone did not significantly attenuate the tumor-induced reduction of thresholds in females, similar to the findings in males. Bortezomib treatment produced profound allodynia, which was effectively reversed by metformin co-treatment in female mice, consistent with the results observed in male mice.

Interestingly, in female mice, both bortezomib and metformin alone significantly reduced tumor growth and weight (Figures 3B,C), in contrast to the findings in male mice where metformin alone did not significantly alter tumor growth. Crucially, metformin co-treatment did not interfere with the anticancer effects of bortezomib in female mice, as demonstrated by the similar tumor growth curves and tumor weights between the bortezomib and bortezomib + metformin groups. These findings further support the potential of metformin as an adjuvant therapy for managing CIPN without compromising the anticancer efficacy of the chemotherapeutic agent in both male and female mice.

The effects of targeting aerobic glycolysis on bortezomib-induced neuropathic pain and tumor growth were also investigated in female mice with LLC implantation (Figure 4). As observed in male mice, bortezomib treatment significantly increased tactile allodynia compared to vehicle-treated female mice (Figure 4A). Co-treatment with either DCA or oxamate effectively attenuated bortezomib-induced tactile allodynia in female mice.

Treatment with bortezomib, DCA, oxamate, or a combination of bortezomib with either DCA or oxamate significantly reduced both tumor volume and weight compared to vehicle-treated female mice (Figures 4B,C). Notably, the anticancer effects of bortezomib were maintained when co-administered with either DCA or oxamate in female mice, as seen in male mice. These findings further support the potential of targeting aerobic glycolysis with DCA or oxamate to complement the anticancer efficacy of bortezomib in both male and female tumor-bearing mice.

In conclusion, the results demonstrate that metformin and targeting aerobic glycolysis with DCA or oxamate can effectively alleviate chemotherapy-induced neuropathic pain without interfering with the anticancer effects of bortezomib in both male and female mice bearing LLC tumors. While some differences were observed between male and female mice, particularly in the effects of metformin alone on tumor growth, the overall findings highlight the potential of metformin and inhibiting aerobic glycolysis as promising therapeutic strategies for managing CIPN in both sexes.

### Sex differences in LLC tumor growth

**Figure 5:**
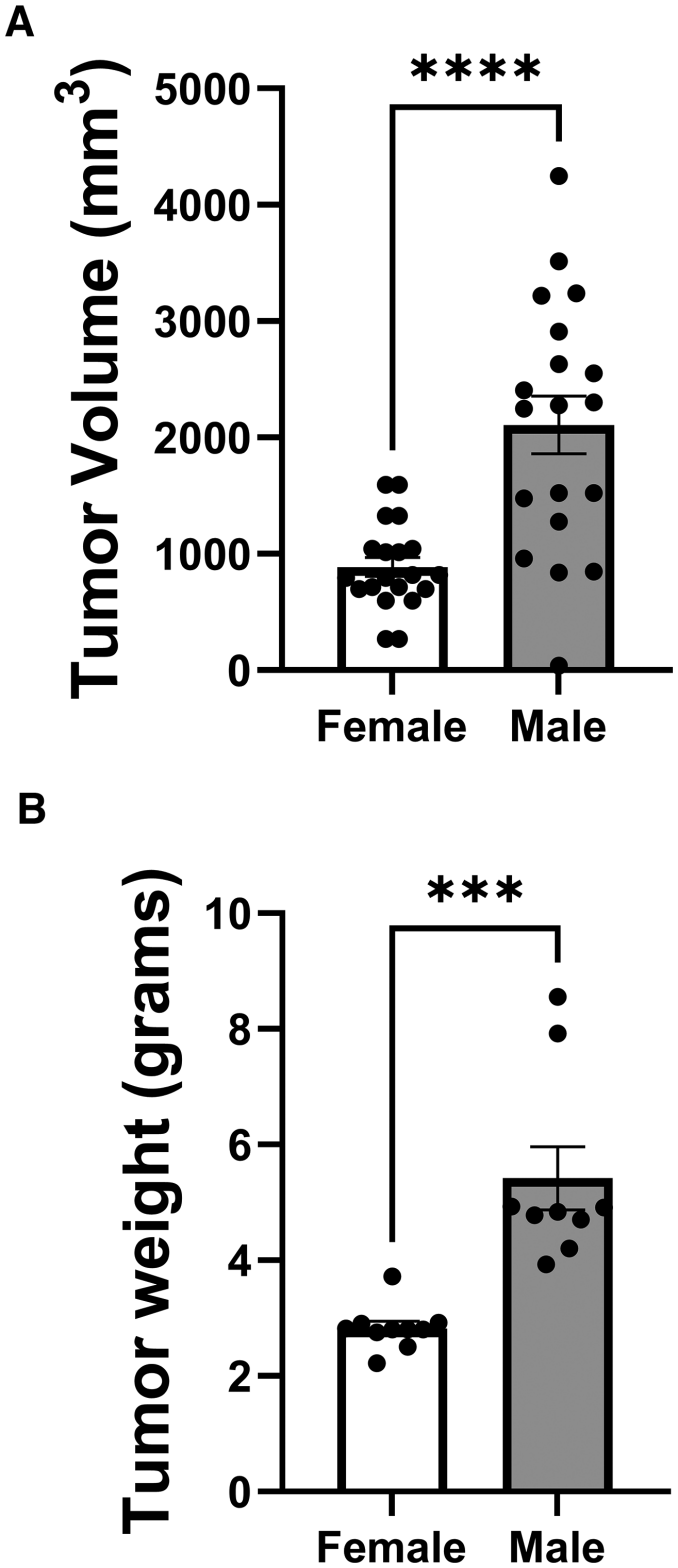
Sex differences in LLC tumor growth. (**A**) Tumor volume measured on day 14 post-inoculation. (**B**) Tumor weight measured on day 16 post-inoculation. (*n* = 10 mice per group). (***p < 0.001, ****p < 0.0001, female vs. male).

To investigate potential sex differences in LLC tumor growth, we compared tumor volume and weight between female and male mice (Figure 5). Tumor volume measurements on day 14 post-inoculation (Figure 5A) revealed a striking sex difference, with male mice exhibiting significantly larger tumor volumes compared to female mice. The mean tumor volume in males was approximately 2.5-fold higher than in females, indicating a substantially faster tumor growth rate in male mice. Similarly, tumor weight data on day 16 post-inoculation (Figure 5B) showed a significant sex difference, with male mice having markedly heavier tumors compared to female mice. The mean tumor weight in males was nearly double that of females, further confirming the accelerated tumor growth in male mice. These findings demonstrate a clear sex disparity in LLC tumor growth, with male mice exhibiting significantly larger and heavier tumors compared to their female counterparts. This sex difference suggests that intrinsic biological factors, such as hormonal influences or sex-specific immune responses, may play a role in modulating tumor growth in this NSCLC mouse model. Further research is needed to elucidate the underlying mechanisms contributing to these observed sex differences in tumor growth.

### Sex differences in the effects of oxamate, metformin, and DCA on bortezomib-induced neuropathic pain and tumor growth

**Figure 6:**
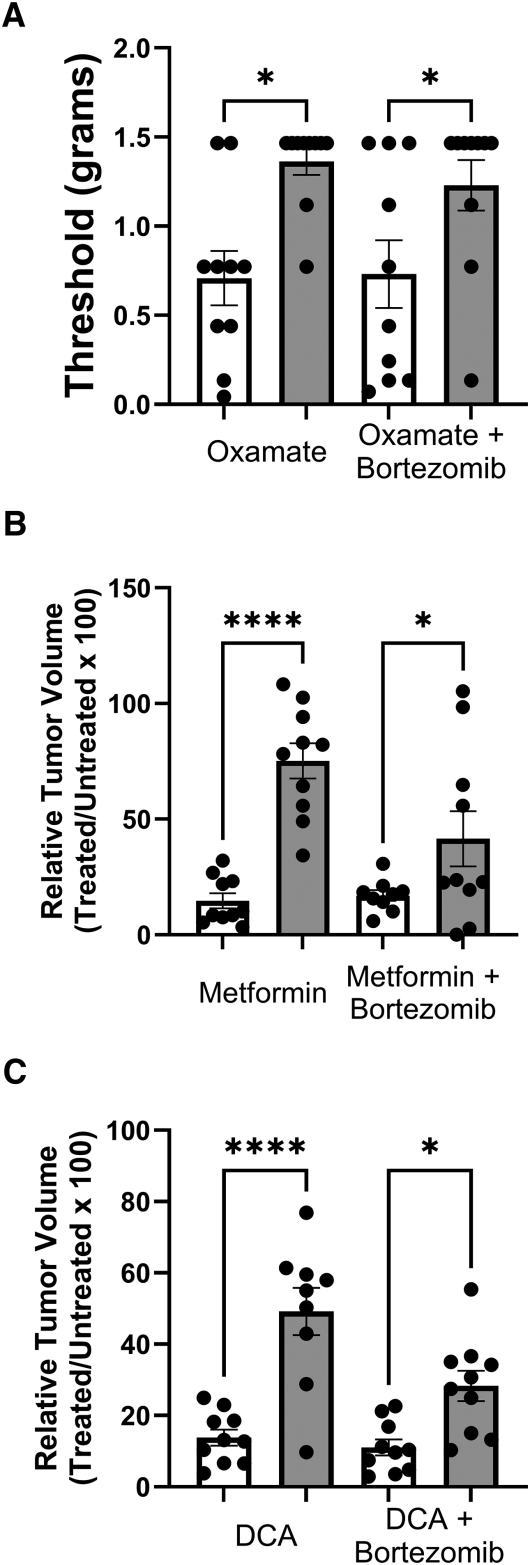
Comparison of tactile allodynia and tumor growth in female (white bars) and male (grey bars) mice. (**A**) Tactile allodynia measured by Von Frey filaments on day 14 post-inoculation and treatment with oxamate and bortezomib. (**B**) Volume of tumors on day 14 in mice treated with metformin and bortezomib. Data shown as percentage of the treated group relative untreated control within each sex. (**C**) Relative tumor volume on day 14 post-inoculation and treated with DCA and bortezomib. (*n* = 10 mice per group). (*p < 0.05, ****p < 0.0001, female vs. male).

Tactile allodynia assessment in mice treated with oxamate and bortezomib (Figure 6A) revealed a sex difference in the efficacy of oxamate in alleviating tumor- or bortezomib-induced neuropathic pain. Oxamate treatment resulted in significantly higher tactile thresholds in male mice compared to female mice, indicating that oxamate may be more effective in reducing LLC- or bortezomib-induced allodynia in males than in females. This finding suggests that sex-specific factors may influence the analgesic efficacy of oxamate in the context of cancer- or bortezomib-related neuropathic pain.

Interestingly, tumor volume measurements in mice treated with metformin and bortezomib (Figure 6B) revealed a contrasting sex difference. Female mice exhibited a significantly lower tumor volume when treated with metformin alone or in combination with bortezomib compared to their male counterparts. This finding suggests that metformin may be more effective in reducing tumor growth in females than in males, highlighting the potential role of sex-specific factors in modulating the anticancer efficacy of metformin.

Consistent with the observations made with metformin, mice treated with DCA and bortezomib (Figure 6C) also displayed a sex difference in tumor volume reduction. DCA treatment, either alone or in combination with bortezomib, led to a significantly lower tumor volume in female mice compared to male mice. This result further supports the notion that sex may influence the efficacy of metabolic interventions, such as DCA, in reducing tumor growth.

In summary, the results presented in Figure 6 demonstrate notable sex differences in the effects of oxamate, metformin, and DCA on bortezomib-induced neuropathic pain and tumor growth. While oxamate appears to be more effective in alleviating tumor- or bortezomib-induced allodynia in male mice compared to females, metformin and DCA seem to be more effective in reducing tumor growth in female mice than in males. These findings underscore the importance of considering sex as a biological variable in preclinical studies investigating potential therapeutic interventions for managing CIPN and cancer treatment. Further research is warranted to elucidate the underlying mechanisms contributing to these sex differences and to optimize treatment strategies based on sex-specific factors.

## Discussion

**Figure 7:**
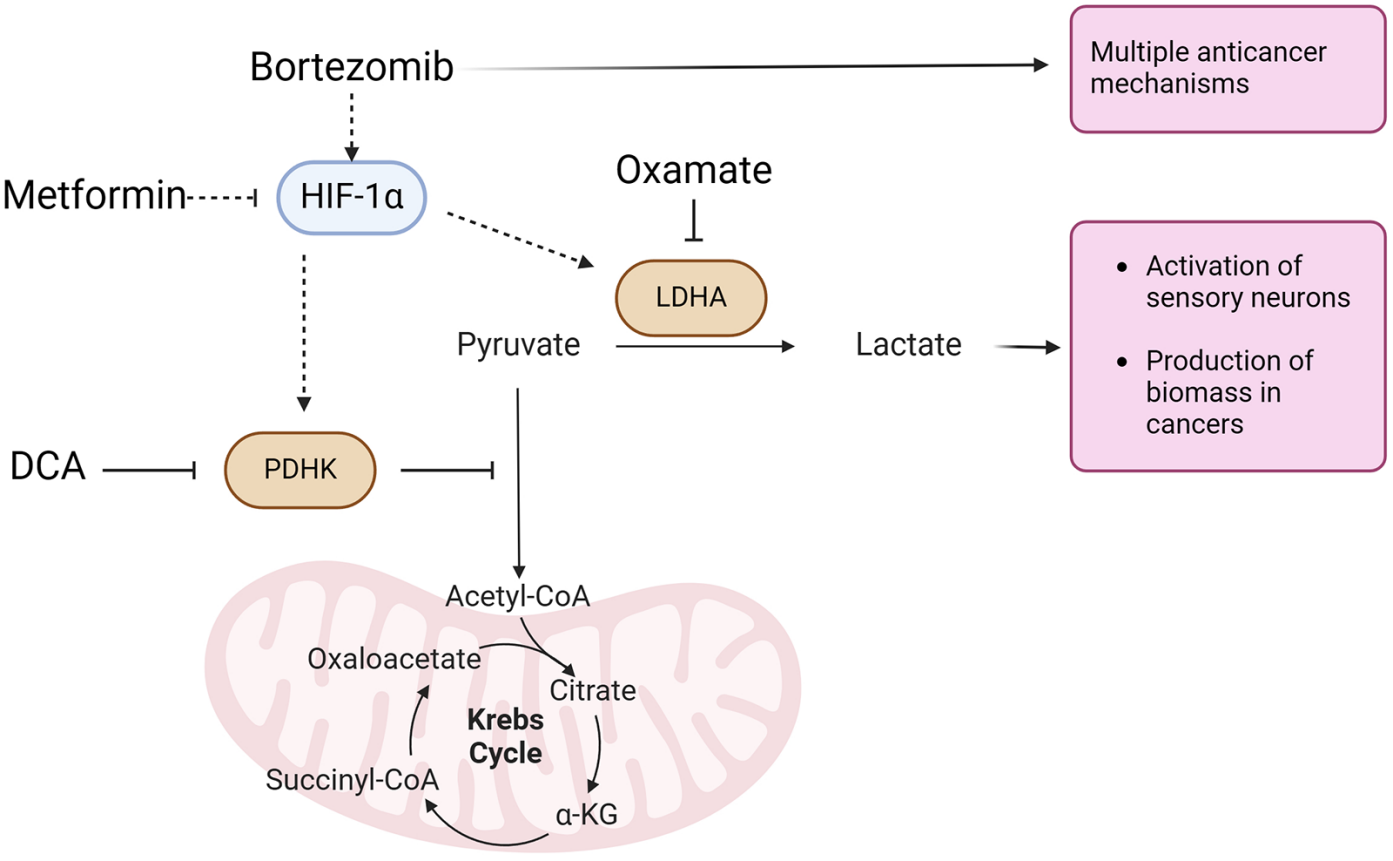
Aerobic glycolysis is the most common metabolic phenotype of cancer cells. Bortezomib, a proteasome inhibitor with multiple anticancer activities, stabilizes HIF-1*α* expression in sensory neurons. HIF-1*α* induces the expression of pyruvate dehydrogenase kinase (PDHK) and lactate dehydrogenase A (LDHA). PDHK inhibits the mitochondrial oxidation of pyruvate, which is subsequently converted to lactate by LDHA. Lactate, along with a proton, is extruded from cells, leading to the activation of sensory neurons driving bortezomib-induced neuropathic pain. Lactate extrusion in cancer cells promotes cancer growth. Conversely, metformin attenuates HIF-1α expression. Dichloroacetate (DCA) and oxamate inhibit PDHK and LDHA, respectively, resulting in the attenuation of both nociceptor hyperexcitability and cancer growth.

The present study investigated the effects of targeting metabolic pathways on bortezomib-induced neuropathic pain and tumor growth using a LLC implanted mouse model, while also exploring potential sex differences in the efficacy of metabolic interventions in alleviating pain and reducing tumor growth. Our findings demonstrate that metformin and aerobic glycolysis inhibitors, such as DCA and oxamate, can effectively attenuate bortezomib-induced neuropathic pain without compromising the anticancer efficacy of bortezomib in both male and female tumor-bearing mice (Figure 7).

A key finding of our study is the development of a paraneoplastic neuropathy-like phenotype in the LLC mouse model, as evidenced by the reduction in tactile thresholds in both male and female mice bearing tumors. This observation suggests that the LLC mouse model may serve as a valuable tool for studying paraneoplastic neuropathy, a rare but devastating condition in cancer patients that greatly impacts their quality of life. Paraneoplastic neuropathy occurs as a result of the immune system's response to cancer. It's part of a group of disorders known as paraneoplastic neurological syndromes, which can affect various parts of the nervous system (32, 33). Further research is needed to validate the utility of this model in investigating the underlying mechanisms of paraneoplastic neuropathy and developing targeted therapeutic interventions.

Interestingly, we observed sex-specific responses to metabolic interventions, with oxamate being more effective in alleviating tumor- and bortezomib-induced allodynia in males compared to females. This finding suggests that the mechanisms underlying CIPN may differ between sexes and that targeting specific metabolic pathways may have sex-dependent effects on pain relief. The sex differences observed in the efficacy of oxamate in alleviating bortezomib-induced neuropathic pain may be related to the sexually dimorphic involvement of innate immune signaling, particularly through Toll-like receptor 4 (TLR4), in the development of neuropathic tactile hypersensitivity. Previous studies have shown that TLR4 antagonism reduces neuropathic pain more effectively in male rodents compared to females (34–37). Furthermore, lactate has been demonstrated to potentiate TLR4 signaling and downstream NF-*κ*B-dependent inflammatory gene expression in macrophages (38). Given that oxamate inhibits lactate dehydrogenase, thereby reducing lactate production, it is plausible that oxamate may indirectly attenuate TLR4 signaling by decreasing lactate levels. Consequently, the greater efficacy of oxamate in reducing bortezomib-induced allodynia in males could be attributed to the more prominent role of TLR4 signaling in mediating neuropathic pain in males compared to females. These findings highlight the importance of considering sex as a biological variable in preclinical and clinical studies investigating potential therapeutic interventions for managing CIPN. Further research is needed to elucidate the underlying mechanisms contributing to these sex differences and to identify sex-specific targets for the development of more effective and personalized treatments for CIPN.

In addition to the sex differences in the efficacy of metabolic interventions for treating bortezomib-induced neuropathic pain, our results also revealed striking sex differences in LLC tumor growth, with male mice exhibiting substantially larger and heavier tumors compared to their female counterparts. This finding aligns with the growing body of evidence demonstrating sex differences in cancer incidence, progression, and survival across many cancer types. These differences are not limited to reproductive cancers, suggesting that broad biological differences between males and females impact cancer development (39–41). The sex disparity in tumor growth observed in our study underscores the importance of considering sex as a biological variable in preclinical and clinical studies investigating cancer biology and treatment.

In addition to differences in tumor growth, we observed sex-specific responses to treatment with metformin and DCA showing greater efficacy in reducing tumor growth in females. These findings suggest that targeting metabolic pathways may have sex-dependent effects on tumor progression. Sex differences in fundamental biological processes, such as DNA repair, immunity, and cellular metabolism, have been shown to translate into sex differences in cancer development and progression. Sex differences in immunity may play a crucial role in shaping the tumor microenvironment and response to therapy. Females generally mount stronger innate and adaptive immune responses compared to males, which likely impacts anti-tumor immunity and response to therapy. In a mouse model of melanoma, lower tumor growth in females was correlated with greater T-cell infiltration, providing experimental evidence linking sex differences in immune response to cancer progression (39–41). As metabolic interventions can modulate the immune system, it is plausible that the sex-specific effects observed in our study may be related to differences in immune function between males and females.

Our findings highlight the need for further investigation into the mechanistic underpinnings of sex differences in CIPN and emphasize the importance of considering sex as a key variable in both preclinical and clinical studies. To effectively identify sex-specific effects and potential sex-dependent responses to therapy, preclinical studies should be designed to include both sexes and be adequately powered, while clinical trials should stratify patients by sex and analyze outcomes accordingly. By incorporating sex differences into CIPN research and treatment strategies, we can develop more personalized and effective therapies that account for the unique biology of each patient, ultimately improving the quality of life for cancer patients undergoing chemotherapy.

While our study demonstrates the potential of targeting metabolic pathways to alleviate bortezomib-induced neuropathic pain and highlights significant sex differences in the efficacy of metabolic interventions, there are several limitations that should be considered when interpreting the results. One limitation is that the findings are based on a single mouse model of non-small cell lung cancer, the Lewis lung carcinoma model. While this model is widely used and has clinical relevance, the results may not be generalizable to other types of cancers. Additionally, translating these findings to humans may require further validation in clinical settings. Another limitation is that we focused on a specific chemotherapeutic agent, bortezomib, and the efficacy of the metabolic interventions in alleviating CIPN caused by other chemotherapeutic drugs remains to be investigated. Lastly, while we identified sex differences in tumor growth and response to metabolic interventions, the underlying mechanisms contributing to these differences were not elucidated, warranting further investigation to better understand the biological basis of these sex-specific effects.

In conclusion, our study demonstrates the potential of targeting metabolic pathways to alleviate bortezomib-induced neuropathic pain and highlights significant sex differences in the efficacy of metabolic interventions. These findings underscore the importance of considering sex as a critical variable in all aspects of CIPN research. Embracing a sex-inclusive approach is crucial for developing targeted and effective strategies for preventing and treating CIPN in both men and women.

## Data Availability

The raw data supporting the conclusions of this article will be made available by the authors, without undue reservation.

## Supplementary material

Supplementary Figure S1 Effect of bortezomib and metformin on tactile allodynia and tumor volume in male mice with LLC tumors implanted in the back. (**A**) Tactile allodynia assessed using von Frey filaments at baseline (BL) and on days 7, 9, 11, and 14 post-implantation. (**B**) Tumor volume measured on the indicated days. (*n* = 10 mice per group). Asterisks indicate significant differences between the vehicle-treated group and other treatment groups (**p* < 0.05, ***p* < 0.01, ****p* < 0.001, *****p* < 0.0001).
