# Supplementary figures and images for: Targeting metabolic pathways alleviates bortezomib-induced neuropathic pain without compromising anticancer efficacy in a sex-specific manner
Source: Front Pain Res (Lausanne). 2024 Jun 24;5:1424348. doi: 10.3389/fpain.2024.1424348 (PMC11228363; doi:10.3389/fpain.2024.1424348)

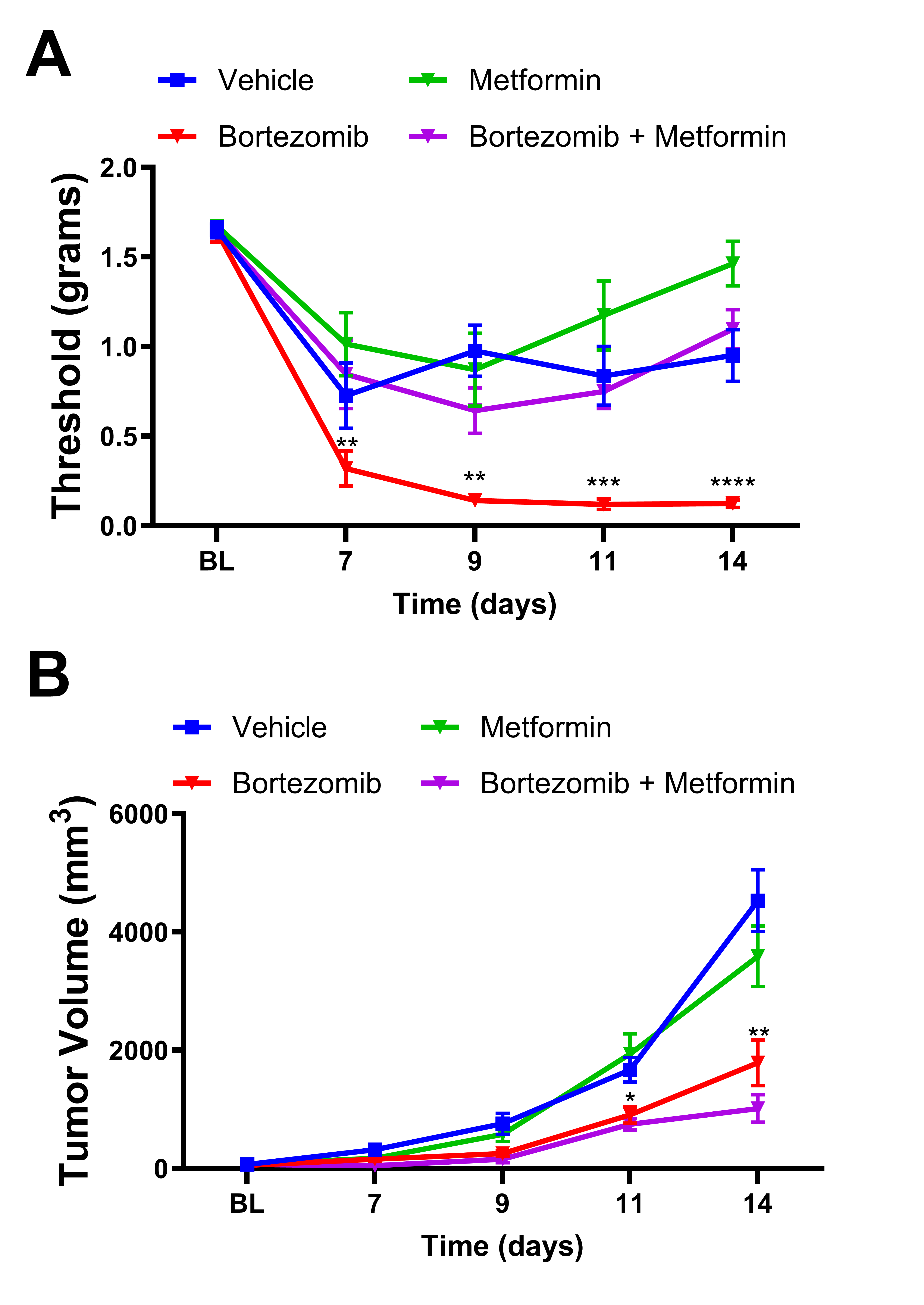

Supplement: Supplementary Figure S1 — Effect of bortezomib and metformin on tactile allodynia and tumor volume in male mice with LLC tumors implanted in the back. (A) Tactile allodynia assessed using von Frey filaments at baseline (BL) and on days 7, 9, 11, and 14 post-implantation. (B) Tumor volume measured on the indicated days. (n = 10 mice per group). Asterisks indicate significant differences between the vehicle-treated group and other treatment groups (*p < 0.05, **p < 0.01, ***p < 0.001, ****p < 0.0001). [file Image1.tif]
